# Different configurations of the two-step floating catchment area method for measuring the spatial accessibility to hospitals for people living with disability: a cross-sectional study

**DOI:** 10.1186/s13690-021-00601-8

**Published:** 2021-05-22

**Authors:** Behzad Kiani, Alireza Mohammadi, Robert Bergquist, Nasser Bagheri

**Affiliations:** 1 Department of Medical Informatics, School of Medicine, Mashhad University of Medical Sciences, Mashhad, Iran; 2 Department of Geography and Urban Planning, Faculty of Social Sciences, University of Mohaghegh Ardabili, Ardabil, Iran; 3 Ingerod, Brastad, Sweden (formerly with the UNICEF/UNDP/World Bank/WHO Special Programme for Research and Training in Tropical Diseases, World Health Organization), Geneva, Switzerland; 4 Visualization and Decision Analytics (VIDEA) lab, Centre for Mental Health Research, Research School of Population Health, College of Health and Medicine, The Australian National University, Canberra, Australia

**Keywords:** Geographical information systems, GIS, Hospital, People living with disability, Spatial accessibility, 2SFCA, Urban area

## Abstract

**Background:**

Poor spatial accessibility to hospital services is associated with higher morbidity and mortality rates among people living with disability. Improved methods to evaluate spatial accessibility are needed. This study measured the potential spatial accessibility of people living with disability by applying four configurations of the two-step floating catchment area (2SFCA) method to recommend the best model for use in health services research.

**Methods:**

2SFCA and an enhanced version (E2SFCA) were used to measure hospital accessibility for people living with disability. We also developed and embedded a non-spatial severity index into the two 2SFCA models. We used 16,186 records of people living with disability experience to evaluate the methodological performance across 68 neighbourhoods of the city of Ahvaz, located in south-western Iran. The models’ performance were measured through correlation of the four accessibility scores with the distance to closest hospital for each neighbourhood centroid.

**Results:**

Among the four models used to measure spatial accessibility, the E2SFCA integrated with the severity index displayed the best performance. Most people with disabilities lived in neighbourhoods located in the South-western and central areas of the city. Interestingly, south-western neighbourhoods had poor hospital accessibility score and were identified as unmet need areas for access to health services.

**Conclusions:**

Inclusion of the severity factor in the E2SFCA improved access measurements. Identifying areas with poor levels of hospital accessibility can help policymakers design tailored interventions and improve accessibility to hospital-based care in urban settings for people living with disability.

**Supplementary Information:**

The online version contains supplementary material available at 10.1186/s13690-021-00601-8.

## Background

Spatial accessibility to hospitals is a challenge for people living with disability, and therefore accounted for an important and integral component of universal health care access [1]. Of 1.5 million (1.8%) people living with disabilities in Iran, 74% live in cities [2], which emphsizes this problem [3–6]. In Iran, hospitals act as general and special care facilities, as well as the first level of referral from prehospital health care services. However, rapid urbanisation has resulted in an unequal distribution of hospitals in urban areas [7–11]. Large inequity in hospital resources and services can exacerbate disparities in health outcomes and quality of life [12], especially for people living with disability.

Accessibility to healthcare is an important dimension to achieve health care for all [13, 14]. The United Nations Convention on the Rights of People with Disabilities [15], the Millennium Development Goals (MDGs) Report 2010 [16], Sustainable Development Goals (SDGs) [17], and the UN-Habitat New Urban Agenda [18] all emphasise the need to identify and eliminate barriers to equal access to health care facilities, particularly for people living with disability in urban areas. Accessibility explains the relative ease by which activities or services can be reached from a given location [19]. Accessibility to hospitals has two main components, spatial and non-spatial [20]. The spatial component refers to locational information such as the number of hospitals in demand catchments and drive times from patients’ homes to the nearest hospital [21]. Non-spatial factors relate to demographic variables, times data and patients’ characteristics. For example, the severity of individuals’ impairment is particularly pronounced in affecting accessibility. Spatial accessibility also is categorized into potential spatial accessibility (PSA) and revealed spatial accessibility (RSA). PSA refers to the probable usage of a service by a needy population in space and time, which is commonly examined based on the evaluation of an existing system of service delivery [22], while RSA demonstrates the actual use of a service, which may be reflected by the frequency of visit to services or individuals’ satisfaction level of using the services [23].

The study of accessibility to healthcare facilities has had a long history in geographical research [24] and different approaches have been applied in assessing PSA including distance to the nearest provider, average distance to a set of providers, and gravitational models of provider influence [25]. The two-step floating catchment area (2SFCA) model was developed by Luo & Wang [26] to examine physician-shortage areas in Illinois, USA. This method addresses two general assumptions such as equal access within a catchment area and no access outside it (binary relation). Luo & Qi [27] noted the limitations of 2SFCA method and developed the enhanced two-step floating catchment area (E2SFCA) method by assigning geographical weights in both steps of the calculation process to differentiate travel-time zones, thereby accounting for distance-decay to maintain theoretical association with the gravity model. The gravity model is the most common example of spatial interaction modelling. The model assumes that the influence of phenomena on each other varies inversely with the distance between them [28]. It is easy to operationalize E2SFCA measure and the results are simple and easy to interpret [29].

While there is an increasing number of studies on the assessment of access to the health care and medical services for all population in urban areas [30–35], little research has relatively focused on measuring PSA to hospitals for people living with disabilities. This study aimed to measure PSA by developing and embedding a disability severity measure in both the 2SFCA and E2SFCA methods to find out if this could improve the outcome measurement. While it was assumed that the combination of E2SFCA and the severity index as a non-spatial factor would best reflect the PSA to hospitals, the primary research question was to find out which methodological configuration would be preferable.

## Methods

### Setting

**Fig. 1:**
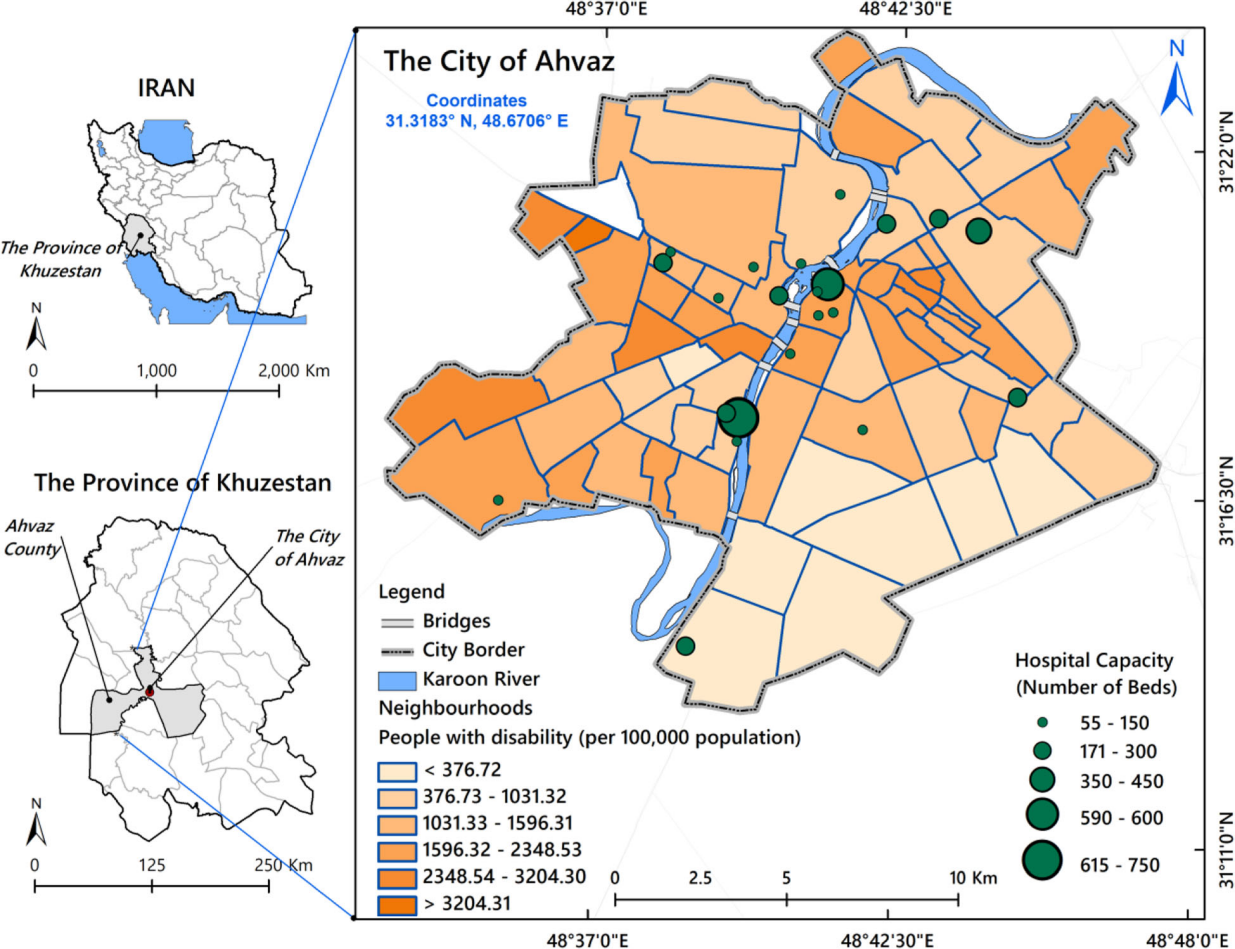
Map of Ahvaz neighbourhoods showing disability rates and hospital distribution by capacity (number of beds)

This cross-sectional study was conducted in 2020 in Ahvaz, the capital city of Khuzestan Province in Iran. Ahvaz’s population has over 1,186,000 inhabitans and covers an area of 258 km^2^ with a average population density of 7771 per km^2^ [36] (Fig. 1). In this research, neighbourhood was the spatial unit in all analyzes. On average, each neighbourhood covers an area of about 4 km^2^ and has an average population of 17,000 people.

### Data/participants

In this study, “people living with disability” refers to those people who live with one or more physical, intellectual, visual, hearing, speaking and mental impairments. Disability data were obtained from the Welfare Organization of Khuzestan Province in 2020 [37]. No sample was taken and all disabled people (*n* = 16,186) were included in the study. Demographic data of the 68 neighbourhoods came from 2016 census blocks [38] and were joined to the neighbourhood spatial layer (as polygon features). Hospitals locations data (as point feature) were obtained from Ahvaz Municipality [36] and they were updated using the outage management system (OMS) online dataset [39]. The addresses of the people living with disability were geocoded and then spatially joined to the neighbourhood layer. The hospital capacity was represented by the number of beds per hospital.

### Spatial statistics

#### Network analysis

In order to measure the demand locations, we followed Luo and Wang [26] approach using disability-weighted centroids and calculated by centroid tool in ArcGIS, version 10.7.1 (ESRI, Redlands, CA, USA). The network analysis dataset included neighbourhoods centroids as origins points, hospitals as destinations and streets by hierarchy. Topology checks were performed to resolve connectivity issues. One important parameter in measuring the spatial accessibility is the drive distance between the supply and demand (hospital and home) locations [30]. Using a travel-time threshold (d) of 5 min in the 2SFCA calculation, an acceptable spatial distance to hospitals in Iran’s metropolitan areas has been estimated at 1–1.5 km travel-distance (0.621–0.932 mile) or at 5–10 min travel-time [40]. The estimated 5 min travel-time buffer for each neighbourhood’s centroid and hospitals service area zones was derived from the ArcMap Network Analyst tool. Based on the hierarchy and speed category within the road network, the standard speed was set at 80 km/h for expressways, 60 km/h for main roads, 40 km/h for secondary roads, and 30 km/h for local roads.

Four methods were used to measure the PSA to hospitals among people living with disability in Ahvaz based on the calculated distances as follows:

1. The 2SFCA method:

An access score ratio computed for each neighbourhood using the 2SFCA approach. In step 1, following previous studies [24, 35] we looked for the location *k* of all people living with disability within the threshold travel-time *d*_*0*_ of 5 min from hospital *j* (i.e. the catchment of this hospital location) and computed the bed-to people living with disability ratio *R*_*j*_ within the catchment area by eq. 1 provided in Additional file 1.

In step 2, we looked for all hospital locations (*j*) for each neighborhood centroid *i* within the threshold travel-time *d*_*0*_ of 5 min from location *i* (i.e. the catchment area) and summed up the bed-to-population ratios *R*_*j*_ (calculated in step 1) at these locations by eq. 2 provided in Additional file 1.

**Fig. 2:**
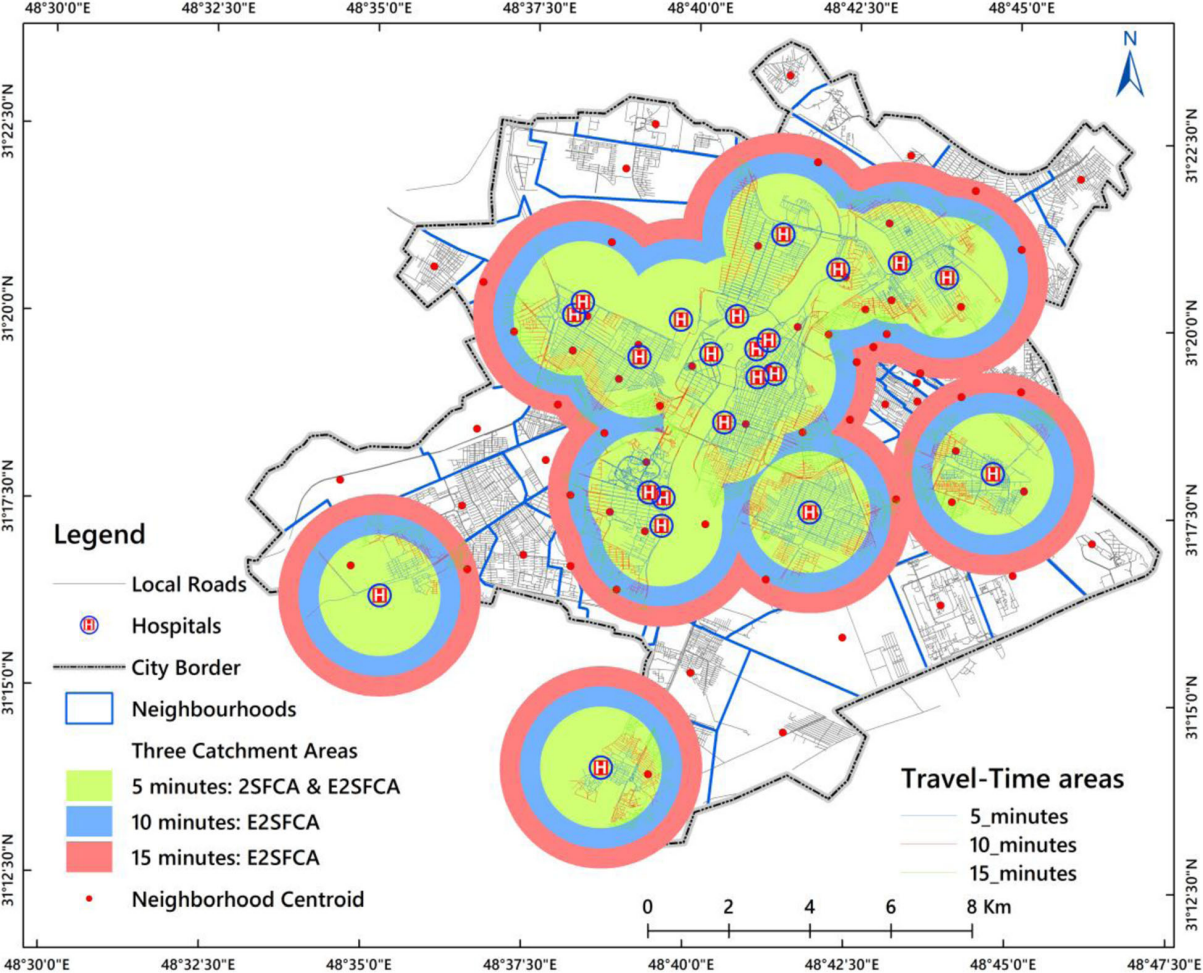
The three catchment areas used with the neighbourhood unit for calculating the PSA

2. The E2SFCA method:

Since the 2SFCA method did not consider distance decay effect in calculating PSA, it led to unrealistic estimates of the access scores. To overcome this limitation, we applied the E2SFCA method introduced by Luo et al. [27, 41] and deployed in our setting as follows:

In step 1, catchment areas set at 5, 10, and 15-min drive time to hospital location *j* were used, i.e. catchments 1, 2 and 3, respectively, as it can be seen in Fig. 2. We searched all people living with disability population locations (*k*) within the threshold travel-time zone (*D*_*r*_) from location *j* (i.e. catchment area *j*), and computed the weighted bed-to-population ratio *R*_*j*_ within the catchment area using eq. 3 provided in Additional file 1. Following previous studies [11, 24, 41, 42], in this equation, *W*_*r*_ represents the distance weight for the *r*^*th*^ travel-time catchment calculated by the Gaussian function. The weights set (1.00, 0.68, 0.22) were used for capturing the distance decay of access to the hospital *j*.

In step 2, we searched all stations locations (*j*) for those living with disability in neighborhood *i*, within the 15-min travel-time catchment from neighbourhood *i* (i. e. catchment area *i*) and summed up the bed-to population ratios *R*_*j*_ (calculated in step 1) at these neighbourhoods by eq. 4 provided in Additional file 1. The same distance weights derived from the Gaussian function used in step 1 were applied to different travel-time zones to account for the distance decay.

3. The disability-severity 2SFCA approach:

People living with disability have diffenet needs and the level of accessibility to health care based on the type and degree of impairment and disability [5]. To integrate severity of disability as a significant non-spatial factor of accessibility into our access measure, we included the severity as weights in the accessibility formula to measure the effect of severity on the PSA score. We set the weight for each severity degree category based on real registered data as follows: 1). Mild; 2). Moderate; 3). Severe; and 4). Very severe disability. After that, for each neighbourhood, the number of disabled people was multiplied by the severity weight. By integrating the severity weights into 2SFCA method, the PSA could be updated as follows;

In step 1, we searched all people living with disability locations (*k*) for each hospital *j* within the threshold travel-time *d*_*0*_ of 5 min from the hospital (i.e. the catchment of this hospital location) and calculated weighted population by multiplying the associated weights with the number of people living with disability in each severity category. Then the weighted bed-to population ratio *R*_*j*_ within the catchment area was calculated by eq. 5 provided in Additional file 1.

In step 2, we searched all hospital locations (*j*) for each neighbourhood centroid *i* within the threshold travel-time *d*_*0*_ of 5 min from location *i* (i.e. catchment area *i*) and summed up the bed-to-population ratios *R*_*j*_ (calculated in step 1) at these locations as shown in eq. 6 provided in Additional file 1.

4. The disability-severity E2SFCA approach:

This method represents a compounded use of methods no. 2 and 3. In this approach, which used the same equations (Eq. 5 & Eq. 6) as method no. 3, we applied weights for the severity of disability (varying between 1 and 4) to calculate the accessibility measure.

### Visualisation and mapping techniques

**Fig. 3:**
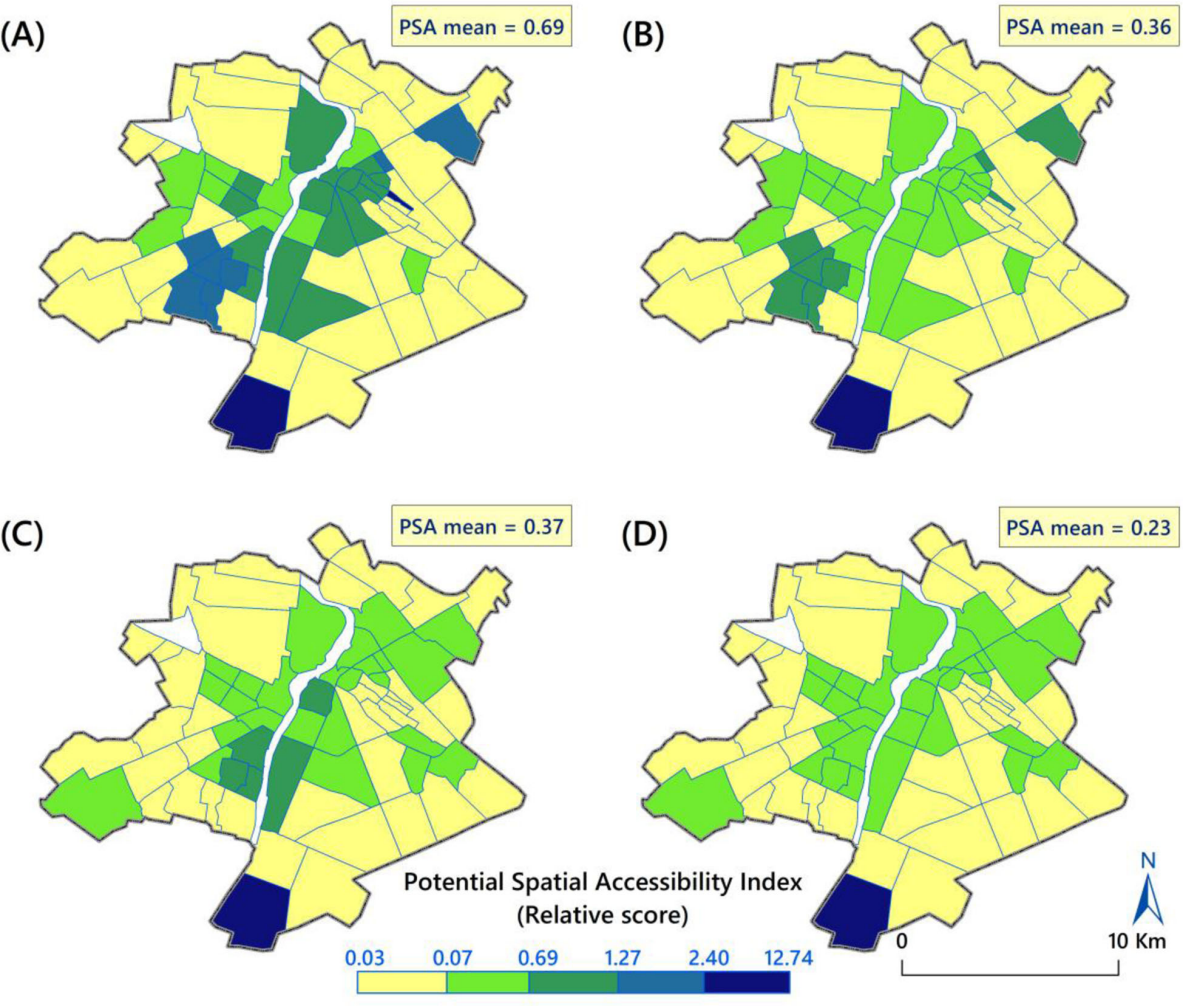
(**a**) PSA for total people with disability experience (2SFCA), (**b**) PSA for severity integrated people living with disability (2SFCA), (**c**) PSA for total people living with disability (E2SFCA), (**d**) PSA for severity integrated people living with disability (E2SFCA)

**Fig. 4:**
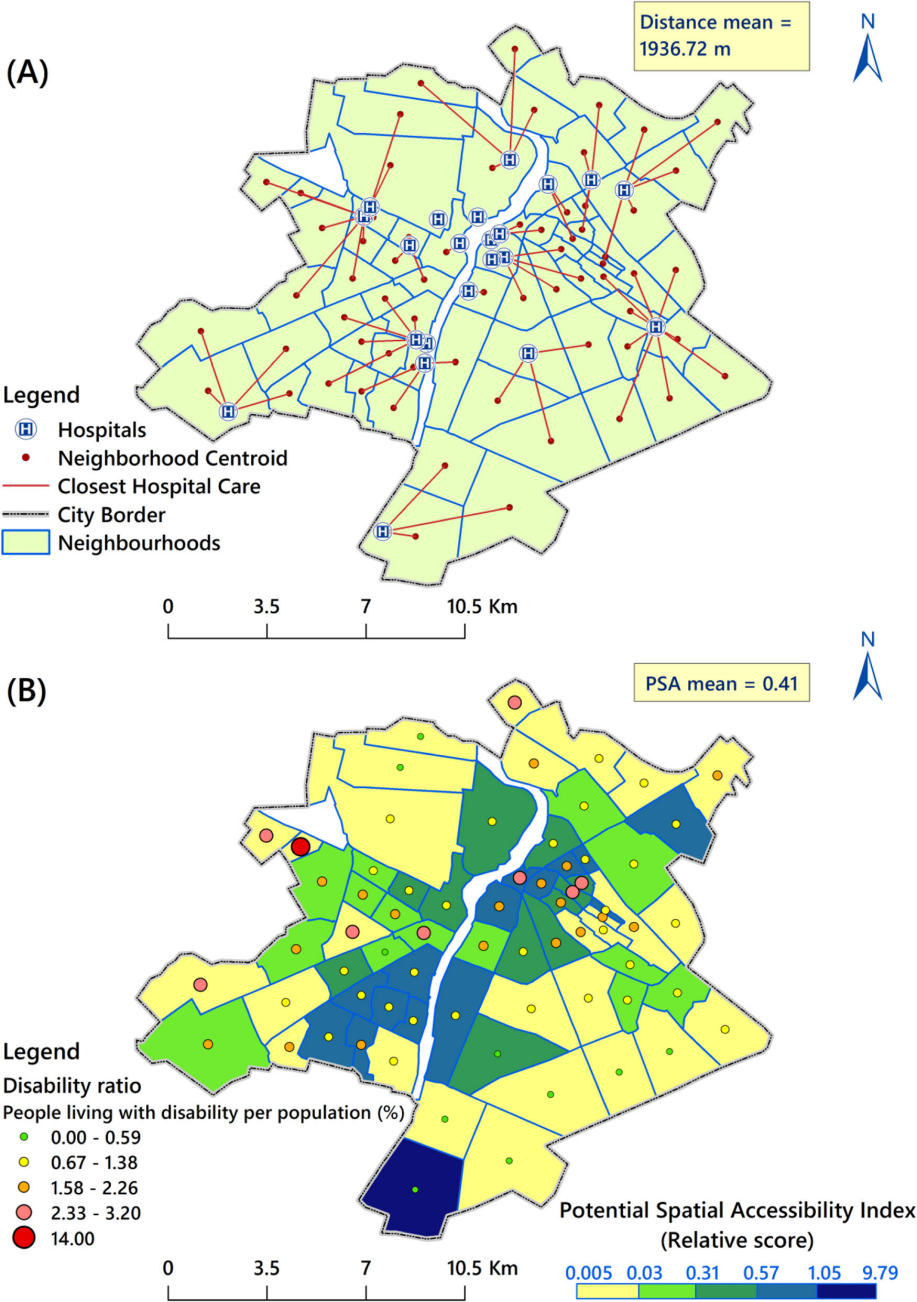
(**a**) Closest hospital to each neighbourhood centroid, (**b**) Overlaid map of the four potential spatial accessibility scores

For the purpose of visual comparison, we categorised the PSA scores into the same intervals for the four methods investigated (Fig. 3). To obtain the relative PSA score, the maps corresponding to the four methods were overlaid by calculating the average of accessibility scores of the four methods for each neighbourhood (Fig. 4b).

### Evaluating performance of the implemented methods to measure PSA to hospitals

We computed the closest distance to hospitals for each geographical neighbourhood centroid for validation of the access measure results (see Fig. 4a). Therefore, for each neighbourhoud, in addition to the four accessibility scores obtained by the four methods, the distance from neighbourhood’s centroid to the closest hospital was also calculated. Further, we used Spearman’s correlation to measure the relationship between the four methods scores with values for the distance to the closest hospital. Spearman correlation coefficient ranged from − 1 to + 1, where + 1 indicates the strongest possible agreement in the same direction, − 1 indicates the strongest possible agreement in the reverse direction, and 0 shows no agreement between two measures.

## Results

### Participants/descriptive data

A number of 16,186 people living with disability resided in urban areas of the city in 2020. The mean age was 33.4 years. Table 1 shows the characteristics of people living with disability. More than 50% of those who were living with disability suffered from severe and very severe disability. As shown in Fig. 1, disability ratio ranged from 0 to 3204 per 100,000 population across neighbourhoods in Ahvaz. The figure shows the spatial distribution of hospitals (*n* = 22) and their capacity, which ranged from 55 to 750 beds.

**Table 1:** Characteristics of the people living with disability in Ahvaz (year 2020)

Disabled population			Disability rate (per 1000)		
*Total*	*Male*	*Female*	*Total*	*Male*	*Female*
16,186	10,362 (64.01%)	5801 (35.98%)	13.33	16.86	9.32
**Characterization by type of disability**					
*Physical*	*Intellectual*	*Hearing & vocal*	*Visual*	*Mental*	
6206 (38.34%)	4864 (30.05%)	2127 (13.14%)	1835 (11.33%)	1154 (7.13%)	
**Characterization by severity of disability**					
*Mild*	*Moderate*	*Severe*	*Very severe*		
1693 (10.46%)	4603 (28.43%)	7010 (43.30%)	2880 (17.79%)		

### Spatial statistic results

Catchment areas of 5, 10, and 15-min drive time to hospitals have been visualised in Fig. 2. As the figure clearly shows, some areas need more than 15 min travel time to reach a health care facility.

Figure 3 shows the PSA to hospital services across neighbourhoods with different access measures for all population and severity-weighted people living with disability. As Fig. 3 shows, PSA scores ranged from 0.03 to 12.74. Based on the results of 2SFCA method (PSA for one person living with disability within 5 min), the average of PSA score was 0.69 for the total number of people living with disability (Fig. 3a) and 0.36 for the severity-integrated people (Fig. 3b).

The results of E2SFCA method showed dissimilarity in PSA scores across the neighbourhoods. Figure 3c shows that average accessibility scores for all people living with disability was 0.37 and Fig. 3d reveals that this value was 0.23 for severity-weighted people living with disability. Based on Fig. 3d, a large proportion of the marginal neighbourhoods had low access ratios (≤ 0.07) and only 20.6% of the neighbourhoods had a greater accessibility score (> 0.23).

As highlighted in all maps (Fig. 3), the peripheral areas of the city had low access to hospital care services compared with the central ones. By applying the severity weights of disability in 2SFCA and E2SFCA methods, the value of accessibility index decreased significantly within all neighbourhoods. In the southern neighbourhoods, the spatial access index was highest among all neighbourhoods (SAI > 12).

### Methodological comparisons

Figure 4a shows the distance between a neighbourhood’s centroid and the closest hospital. The correlation between this distance and the four accessibility scores is shown in Table 2. Although the coefficients indicated a moderate correlation, the access score was improved when the enhanced 2SFCA model with the integrated severity factor was applied (Table 2).

**Table 2:** Correlation coefficient of potential spatial access indices and closest facility distance (CFD)

	People living with disability (all) 2SFCA	Severity – integrated 2SFCA	People living with disability (all) E2SFCA	Severity – integrated E2SFCA
Distance to closest hospital	−0.373*	−0.373*	− 0.888*	−0.889*

*Spearman correlation significant (*P-*value <.05)

Figure 4b shows the overlaid (combined) map of PSA based on average values ranges from 0.005 to 9.79 for each people living with disability in the city. According to this map, the mean of PSA score was 0.41 with a SD of 1.183. Out of the 68 neighbourhoods, 21 (30.9%) had high access ratios (>0.57). As the final map shows, the western areas of the city has low access to the hospital services and also more people living with severe disability.

## Discussion

To the best of our knowledge, this is the first study to integrate the severity of disability dimention into 2SFCA methods to measure PSA to hospitals. The results of E2SFCA method showed that only just under a third of people with disability experience had appropriate PSA to hospitals. This result is in consistent with the findings of Rocha et al. [43] study in Fortaleza, Brazil and Ghasemzadeh et al. in Tehran, Iran [44]. Our findings reveal that a large proportion of people with disability experience have physical impairments that they may need to reach hospitals with a minimum travel time in emergency situation. The study finding also show that people living with disability with a higher severity of impairments have serious obstacles in access to hospital services, especially in potential emergency response times (Fig. 4b). We are aware that even a perfect PSA to hospitals in a particular area would not necessarily lead to better access in reality due to many other confounding factors. However, as indicated by our case study in Ahvaz metropolitan area, the E2SFCA method is more reasonable than the 2SFCA method. It represents a more realistic measure of PSA and the spatial pattern of PSA to hospitals by using distance decay function and applying multi-travel-time approach. In addition, the severity integrated E2SFCA method showed better results compared with E2SFCA method. Therefore, our hypothesis was approved and we thus recommend using the E2SFCA method integrated with the severity index to measure PSA to hospitals for people living with disability in urban areas.

We identified some areas with poor PSA to hospitals in peripheral neighbourhoods of Ahvaz City and our findings are in line with the study of Akuffo et al. [45] in Kumasi, Ghana. The results are also consistent with the findings of Cheng et al. [46] on the imbalance in hospital distribution in Shenzhen, China. Our findings show that the distribution of hospitals in urban neighbourhoods is spatially heterogeneous and unequal. It is necessary to point out that, western neighbourhoods with low accessibility index (low access to hospitals), have also higher number of people living with severe disability and this represents unmet areas for accessibility and future policy interventions could be focused in these areas with poor accessibility to hospitals (Fig. 4)b. A study by Aldersey et al. [47] revealed that wheelchair users seemed to face greater physical barriers to access to health services in cities. The results of the study of Rocha et al. [43] from Fortaleza City in Brazil, revealed that more than 37% of people living with disability, face serious barriers in accessing health care services. Most recent studies reported significant disparities in the access of people living with disability to health care services in Iran’s metropolitan areas [8, 48]. For example, Ghasemzadeh et al. [44] showed that physically disabled people in Tehran, face numerous difficulties in accessing health services. Therefore, as Gleeson study [49], we would suggest to conduct a spatial approach to better highlight and visualise inequality in access to hospital services for people living with disability in urban area.

In this study, as a case study in Ahvaz urban area, we tested four configurations of the 2SFCA method to measure PSA to hospitals for people living with disability. This study approach could provide a foundation for future study of accessibility of people living with disability across the world. The main contribution of this work to accessibility knowledge is adding the severity index as a non-spatial factor into the E2SFCA method.

### Limitations

Our study has some limitations. First, there was no proper traffic information available to embed in the network dataset to conduct multi-mode travel-time based methods. Another limitation was the difficulty to access detailed hospital data, such as the number of physicians or the number of hospitalized people living with disability for each month. Additionally, we did not have any data estimating the flow of people living with disability during day and night.

## Conclusions

Modelling spatial accessibility to hospitals for people with disability at various levels of severity in a metropolitan area is significant. Our study concludes that the integration of disability severity into the E2SFCA method improves the accuracy of estimating accessibility score considerably and it can be taken into account for policy making. Spatial accessibility models should measure the accessibility of hospital services in such a way that all people living with disability would have adequate accessibility to hospital services when it is needed.

## Supplementary Information

**Additional file 1.**

## Data Availability

Raw data on persons live with disability were obtained from the Welfare Organization of Khuzestan Province, Ahvaz. There is no permission to obtain the datasets and they are available from the corresponding author on request.

## Abbreviations

2SFCA: Two-Step Floating Catchment Area
SAH: Spatial Accessibility to Hospitals
SAI: Spatial Accessibility Index
E2SFCA: Enhanced Two-Step Floating Catchment Area
GIS: Geographical Information System
