# Supplementary material for: Different configurations of the two-step floating catchment area method for measuring the spatial accessibility to hospitals for people living with disability: a cross-sectional study
Source: Arch Public Health. 2021 May 22;79:85. doi: 10.1186/s13690-021-00601-8 (PMC8141247; doi:10.1186/s13690-021-00601-8)
Supplement: Supplementary file 1 — Additional file 1. [file 13690_2021_601_MOESM1_ESM.docx]

| $R_{j}=\frac{B_{j}}{\sum_{k} \in\left\{ \left. d_{kj\leq}d_{o} \right\}P_{k} \right.}$ | Eq.1 |
| --- | --- |
| *P_k_* is number of the people living with disability‎ in neighborhood *k* where the centroid falls within catchment *j* (*d_kj_ ≤ d_0_*), *B_j_* the number of beds (capacity) at hospital *j*, and *d_kj_* the travel-time between *k* and *j*. |  |
| $A_{i}^{F}=\sum_{j\in\left\{ \left. d_{ij\leq}d_{o} \right\} \right.} R_{j}$ | Eq.2 |
| $A_{i}^{F}$represents the accessibility of the people living with disability‎ at neighbourhood *i* to hospitals, *R_j_* the bed-to-population ratio at hospital *j* where the centroid falls within the catchment area centred at people living with disability‎ location *i* (ie, *d_ij_ ≤ d_0_*), and d*_ij_* the travel-time between *i* and *j.* |  |
| R_j_$= \frac{B_{j}}{\sum_{k\in\left\{ d_{kj\in D_{r}} \right\}} P_{k}W_{r}}= \frac{B_{j}}{\sum_{k\in\left\{ d_{kj\in D_{1}} \right\}} P_{k}W_{1}+\sum_{k\in\left\{ d_{kj\in D_{2}} \right\}} P_{k}W_{2}+\sum_{k\in\left\{ d_{kj\in D_{3}} \right\}} P_{k}W_{3}}$ | Eq.3 |
| *P_k_* represents the people living with disability‎ in neighbourhood *k* falling within catchment *j* (*d_kj_* ‎∈‎ *Dr*), *B_j_* the number of beds at hospital *j*, *d_kj_* the travel-time between *k* and *j*, and *D_r_* the *r^th^* travel-time zone (*r* $\in$\|1,2, 3\|) within the catchment. |  |
| $A_{i}^{F}=\sum_{j\in\left\{ \left. d_{ij\leq}d_{r} \right\} \right.} R_{j}W_{r}= \sum_{j\in\left\{ \left. d_{ij\leq}D_{1} \right\} \right.} R_{j}W_{1}+ \sum_{j\in\left\{ \left. d_{ij\leq}D_{2} \right\} \right.} R_{j}W_{2}+\sum_{j\in\left\{ \left. d_{ij\leq}D_{3} \right\} \right.} R_{j}W_{3}$ | Eq.4 |
| $A_{i}^{F}$ represents the SAI for the people living with disability‎ in neighborhood *i*, *R_j_* the bed-to-population ratio at hospital location *j* that falls within the catchment centred at neighbourhood *i* (i.e. *d_kj_* ∈‎ *D_r_*), and *d_ij_* the travel-time between *i* and *j*. |  |
| $R_{j}=\frac{B_{j}}{\sum_{k} \in\left\{ \left. d_{kj\leq}D_{o} \right\}\sum_{n} \in\left\{ \left. S \right\} \right.P_{kn}W_{n} \right.}$ | Eq.5 |
| *W* is the weight of disability severity, *P_kn_* the number of people living with disability in each severity category *n* at neighborhood *k* where the centroid falls within catchment *j* (*d_kj_ ≤ d_0_*), *B_j_* the number of beds in hospital *j*, and *d_kj_* the travel-time ‎between *k* and *j*. |  |
| $A_{i}^{F}=\sum_{j\in\left\{ \left. d_{ij\leq}d_{o} \right\} \right.} R_{j}$ | Eq.6 |
| where $A_{i}^{F}$represents the accessibility of the people living with disability (weighted by severity) at neighbourhood *i* to hospitals, *R_j_* the bed-to-population ratio at hospital *j* where the centroid falls within the catchment area centred at people living with disability in location *i* (i.e. *d_ij_ ≤ d_0_*), and d*_ij_*  the travel-time ‎between *i* and *j.* |  |
